# Possible control of acute outbreaks of a marine fungal pathogen by nominally herbivorous tropical reef fish

**DOI:** 10.1007/s00442-020-04697-7

**Published:** 2020-07-12

**Authors:** B. P. Neal, B. Honisch, T. Warrender, G. J. Williams, T. M. Work, N. N. Price

**Affiliations:** 1 Bigelow Laboratory for Ocean Sciences, 60 Bigelow Drive, East Boothbay, ME 04544 USA; 2 School of Ocean Sciences, Bangor University, Anglesey, LL59 5AB UK; 3 US Geological Survey National Wildlife Health Center, Honolulu Field Station, 300 Ala Moana Blvd, Honolulu, HI 96850 USA

**Keywords:** Coral reefs, Crustose coralline algae (CCA), Coralline fungal disease (CFD), Mycophagy, Disease control

## Abstract

Primary producers in terrestrial and marine systems can be affected by fungal pathogens threatening the provision of critical ecosystem services. Crustose coralline algae (CCA) are ecologically important members of tropical reef systems and are impacted by coralline fungal disease (CFD) which manifests as overgrowth of the CCA crust by fungal lesions causing partial to complete mortality of the CCA host. No natural controls for CFD have been identified, but nominally herbivorous fish could play a role by consuming pathogenic fungi. We documented preferential grazing on fungal lesions by adults of six common reef-dwelling species of herbivorous Acanthuridae and Labridae, (surgeonfish and parrotfish) which collectively demonstrated an ~ 80-fold higher grazing rate on fungal lesions relative to their proportionate benthic coverage, and a preference for lesions over other palatable substrata (e.g. live scleractinian coral, CCA, or algae). Furthermore, we recorded a ~ 600% increase in live CFD lesion size over an approximately 2-week period when grazing by herbivorous fish was experimentally excluded suggesting that herbivorous reef fish could control CFD progression by directly reducing biomass of the fungal pathogen. Removal rates may be sufficient to allow CCA to recover from infection and explain historically observed natural waning behaviour after an outbreak. Thus, in addition to their well-known role as determinants of macroalgal overgrowth of reefs, herbivorous fish could thus also be important in control of diseases affecting crustose coralline algae that stabilize the foundation of coral reef substrata.

**Electronic supplementary material:**

The online version of this article (10.1007/s00442-020-04697-7) contains supplementary material, which is available to authorized users.

## Introduction

Primary producers form the foundations of terrestrial and aquatic ecosystems and serve as habitat and food for a plethora of organisms, but disease can threaten the provision of these critical ecosystem services. In particular, outbreaks of fungal infections commonly contribute to species die-offs, biodiversity loss, and declines in ecosystem function and health (Fisher et al. 2012). Loss of plant hosts due to fungal infections can have important ecological and economic ramifications in terrestrial systems (Oerke 2006). For example, wheat rust has reduced yields from food crops for humans (Kolmer 2005) and chestnut blight has destroyed nearly all native trees of that species in North America (Anagnostakis 1987). While fungi are known to comprise a large fraction of plant pathogens in terrestrial ecosystems (Agrios 2005), less is known about the prevalence, interactions, and particularly the dynamics of fungal infections of primary producers in marine environments (Amend et al. 2012; Worden et al. 2015; Scholz et al. 2016). One important group of marine primary producers affected by disease, including by fungal pathogens, is the crustose coralline algae (CCA).

**Fig. 1:**
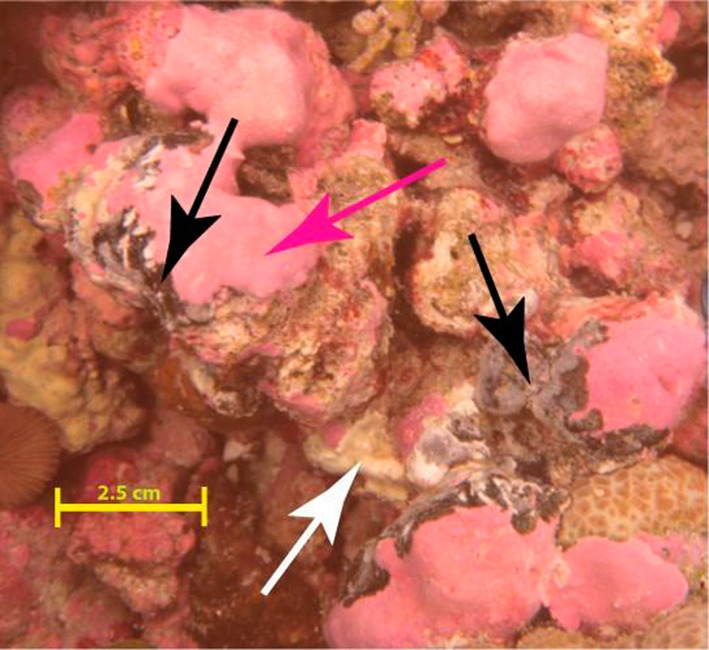
An example of a coralline fungal disease (CFD) lesion on a crustose coralline algal (CCA) host. Black arrows mark active CFD lesions, seen as a black or grey material on healthy *Porolithon onkodes* crust (pink arrow). The white arrow likely indicates an area of recent CFD infection, where the fungus has moved through, leaving dead CCA behind (Photo: T. Work)

CCA are ecologically important to tropical reef systems because some species of CCA provide preferential substrate for settlement of scleractinian coral planulae (Harrington et al. 2004; Price 2010; Fabricius et al. 2017), cement reef structure (Manzello et al. 2008) and contribute to carbon cycling (Glynn and Manzello 2015). Our understanding of the number of diseases impacting CCA is sparse but growing. There are currently six documented types of CCA disease (Littler and Littler 1997; Vargas-Ángel 2010; Williams et al. 2014; Quéré and Nugues 2015), but only two have identified infectious mechanisms associated with the lesions they present (Quéré and Nugues 2015)—coralline lethal orange disease (CLOD) (Littler and Littler 1995) and coralline fungal disease (CFD) (Littler and Littler 1998). The former is thought to be associated with bacteria and causes orange discoloration (Littler and Littler 1995). CFD, the focus of this work, is associated with overgrowth of CCA by fungal hyphae and manifests as continuous or patchy grey-black surficial lesions (Fig. 1). *Porolithon onkodes* is the only currently documented host for this disease (Williams et al. 2014). CFD occurs across the tropical Pacific and has recently been documented in the Indian Ocean, with high densities of lesions reported in the Chagos Archipelago, British Indian Ocean Territory (Williams et al. 2018). CFD distribution is patchy across the region, and temporally dynamic, with greater occurrence of CFD documented in American Samoa and Palmyra Atoll in the Central Pacific (Vargas-Ángel 2010).

Tropical marine reef environments present interesting cases for examining pathogen dynamics and the ecological degradation associated with disease events (Porter 2013). In contrast to terrestrial ecosystems, diseases in marine ecosystems are prone to spread more rapidly due to the potential for distant transport in oceanic currents (McCallum et al. 2003). The widespread rapid loss of Caribbean corals due to disease is one example of this dynamic (Kline and Vollmer 2011, Randall and Van Woesik 2015). Furthermore, reef environments are particularly susceptible to local stressors such as land based pollution (Fabricius 2005) and global stressors such as rising ocean surface temperatures and acidification (Kleypas et al. 2001). Importantly, human activity also may be creating conditions that exacerbate fungal disease in primary producers (Garrett et al. 2015). This is the case with CFD where outbreaks are associated with extended seawater warming events (Williams et al. 2014). Whether the mechanisms responsible for changes in marine disease dynamics are driven by reduction in host immunity, enhancement of pathogen virulence and/or transmission, loss of biological controls suppressing the infection, or a combination of these remains unclear.

In contrast, the factors that influence the pathogenesis of fungal diseases in terrestrial plants are well characterized and numerous chemical and biological control options exist to limit the effects of fungal infection on hosts (Heydari and Pessarakli 2010). The role of mycophagy and associated impacts on primary producers has been described for a range of terrestrial systems, including beetles (Lawrence 1989), marsupials (Claridge and May 1994), small forest mammals (Claridge et al. 1999), primates (Sawada et al. 2014), upland birds (Tanney and Hutchison 2011), and soil mites (Smrž et al. 2016). For example, the mycophagous mite *Orthotydeus lambi* has been tested as an agricultural agent for reducing virulence of powdery mildew on commercial grape foliage and fruit (English-Loeb et al. 1999). Plant diseases caused by fungi have been successfully controlled with: (1) bacteria (Weller 1988; Heydari and Pessarakli 2010); (2) avirulent fungi which are parasitic to other pathogenic fungi (Harman et al. 2004) and; (3) mycophagous mites (English-Loeb et al. 1999).

**Fig. 2:**
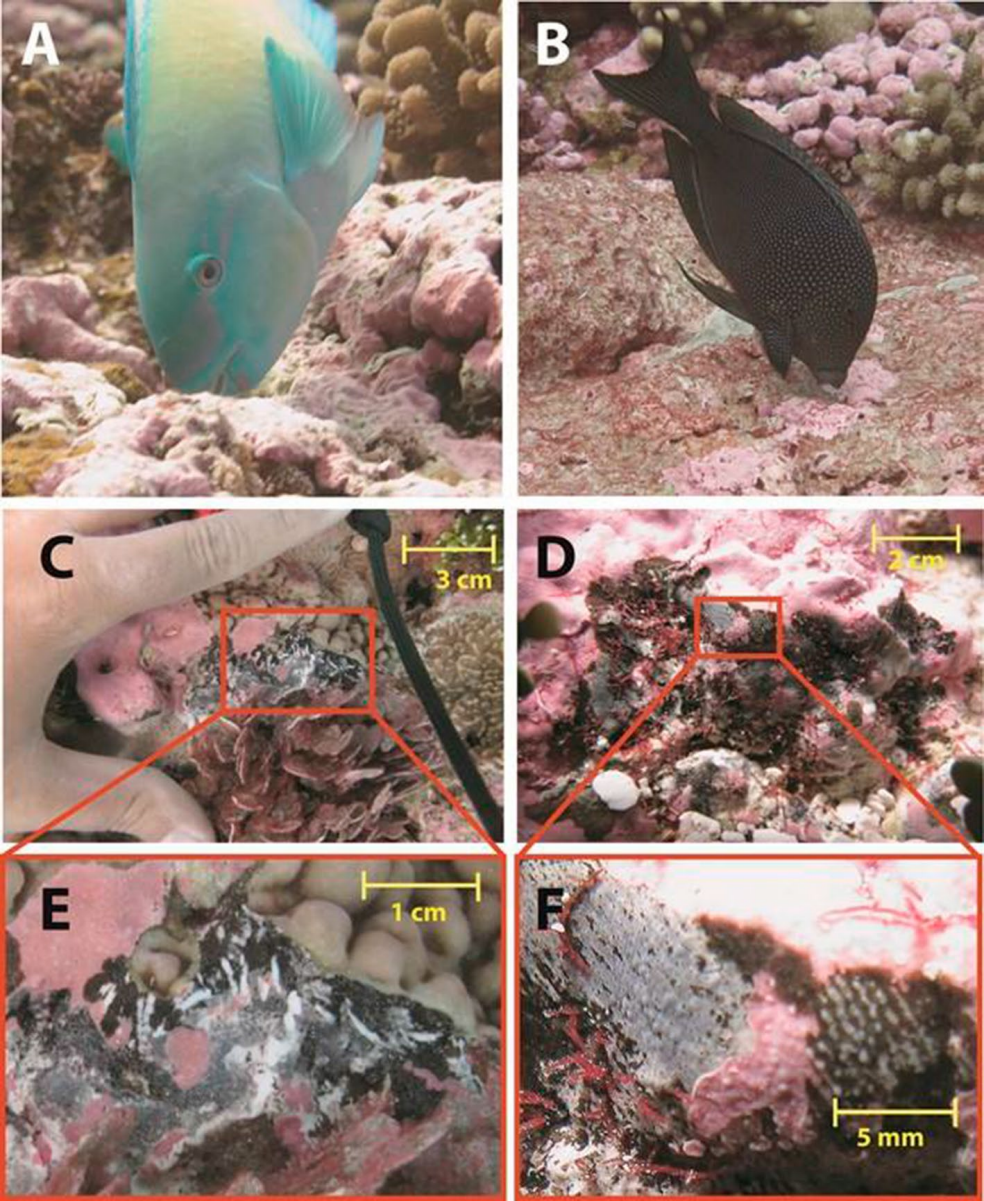
Two examples of the six herbivorous fish species observed grazing on CFD lesions and surface evidence of grazing on CFD lesions. **a***Chlorurus spilurus (*scarine labrid). **b***Ctenochaetus marginatus *(family Acanthuridae). **c** CFD fungal lesion, overlain and partially protected by a CCA encrusted *Halimeda fragilis* with typical visible surface scars caused by bites from scarine labrids). **d** CFD fungal lesion showing fine scale tooth marks indicative of grazing by *Acanthuridae *sp*.***e** Close-up of **c.f** Close-up of **d** (all photos: B. Neal)

Similar mechanisms of biological control for fungal infections may exist in marine ecosystems. For instance, fishes historically described as herbivorous in coral reef systems are now known to consume a varied diet, including macroalgae, turf algae, coral polyps, pelagic and planktonic animal matter, organic detritus and sediments (Wilson and Bellwood 1997; Choat et al. 2002; Wilson et al. 2003), and fish scrapes or bites are commonly seen on a variety of substrates, including CFD lesions (Fig. 2). Fish could, therefore, potentially play a similar role by consuming pathogenic fungi. Targeted consumption of the fungal tissue by any community members, including herbivorous fish, could have direct implications for the etiology, severity, and duration of CFD disease events, but the contribution of this type of interaction to disease dynamics has not been previously tested. To examine the relationship between marine herbivorous fish and the fungal pathogen, and to assess the impacts on tropical CCA from CFD infection, we investigated behavioral interactions of reef fish and fungal lesions using a combination of field surveys and experimental manipulations. Our objectives were: (1) to evaluate whether herbivorous fish manifested a preference for grazing on CFD-affected CCA, and (2) to document progression of CFD on CCA with and without release from grazing by herbivorous fish through the use of experimental fish exclosures.

## Materials and methods

### Site description

**Fig. 3:**
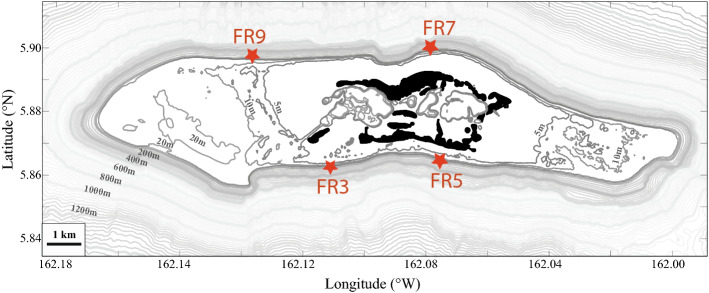
Map of Palmyra Atoll. The four sample sites and names are marked in red. Black area is dry island area, white area is atoll calcium carbonate platform, largely euphotic with hard coral and other shallow marine habitats, and grey contours indicate benthic depths beyond the atoll edge. All CFD, benthic community, and fish survey sites for this study were on the fore reef, in depths from 10 to 30 m

The study site was located on Palmyra Atoll National Wildlife Refuge in the Pacific Northern Line Islands chain, (05° 52′ N, 162° 06′ W) which has been jointly managed by The Nature Conservancy and the US Fish and Wildlife Service since 2001. The atoll saw extensive alterations as a military base during World War II (Collen et al. 2009). However, since its protection, direct anthropogenic impacts have been minimal (Agardy 2004). High live coral cover and good water visibility characterize the outer fore reef, and fish populations around Palmyra are considered nearly pristine (Sandin et al. 2008). Total fish biomass is ca. 250–350 g m^−2^, with herbivores comprising ca. 15–30% (DeMartini et al. 2008). Compared to degraded coral reefs, Palmyra has approximately an order of magnitude greater biomass of herbivorous fishes (Jackson et al. 2014), which are particularly important to maintaining dominance of calcifying (reef-building) organisms in this system, as the atoll naturally lacks large densities of herbivorous echinoids (Hamilton et al. 2014). Four permanent monitoring sites were used in this study, on the fore reef slope to the southwest, southeast, northeast, and northwest of the atoll center, respectively designated FR3, FR5, FR7, and FR9 (Fig. 3). Fieldwork took place from 26-Jun-2016 to 18-Jul-2016, and from 30-Jul-2017 to 20-Jul-2017, following the recent 2015/2016 El Nino event (Brainard et al. 2018).

#### Herbivorous fish grazing preference

To assess herbivorous fish preferences for a particular substrate, we first determined cover of relevant benthic types from habitat and disease surveys and compared the relative abundance of benthic cover strata to independent fish grazing behaviour observations at the same four sites (methods for both habitat surveys and fish observations presented in detail below). As our primary interest was fish behaviour across the system, both benthic cover and fish behaviour data from the four sites were pooled for the entire atoll.

### Benthic community surveys

To characterize benthic habitat, we used Point Intercept Transect (PIT) methods (Hill and Wilkinson 2004) at each of the four sites during 8–16 July 2016. Two isobathic transects were deployed parallel to the reef crest along the 10-m and 15-m depth contours of the fore reef slope. A third transect was deployed perpendicular to the first two, along a depth gradient starting at 25 m depth and continuing to the minimum practicable depth on the reef crest, as limited by swell and surf (~ 5 m depth). Each survey consisted of a pair of divers repeatedly laying out a 25-m transect tape and categorizing substrate at 1-m intervals to one of nine categories (crustose coralline algae, scleractinian corals, octocorals, macroalgae, corallimorphs, dead coral/coral rubble/turf algae, bare sand, and other). Total transect lengths per dive ranged from 77 to 160 m, depending on dive conditions and diver endurance. Percent cover and areal coverage of primary benthic types (including abundance of CCA) for surveyed areas was calculated on an island-wide basis from the PIT observations. Different CCA species present very similar appearances in the field, making species-level identification in situ impractical. Percent cover is thus reported as total percent cover for all CCA species. Given that CFD is only known to infect a single host, our observed CCA cover is larger than the area of possible CFD infection.

### In situ coralline fungal disease (CFD) surveys

The relatively rare and cryptic nature of CFD lesions precluded quantification of the benthic coverage of CFD using point intercepts, so a separate dive team accompanied the benthic cover survey team and closely examined a 2-m wide continuous band transect of the benthos to record the presence of CFD lesions, along with depth and maximum diameter of all lesions. Non-contiguous lesions in clusters were each counted as individual lesions. Lesion area was estimated by calculating the coverage of each lesion as the area of a circle using the largest measured diameter of each lesion. CFD abundance was summarized as both percent cover for the sampled area and also as the number of fungal lesions per square meter of CCA host as calculated from PIT survey data.

The percent cover estimate for CFD is almost certainly a slight overestimation due to the assumption of circularity in the calculation based on maximal measured diameter of a CFD lesion, and the observed irregularity of lesion shapes. However, this marginal overestimation of total lesion coverage was retained in subsequent calculations of feeding preference, because it would ensure a slightly conservative outcome when estimating grazing preference for this category; marginally larger areal coverage estimate for any individual category (such as CFD) will mathematically result in proportionately lower (i.e. conservative) calculation of relative feeding preference.

### Herbivorous fish behavioral observations

For herbivorous fish feeding observations, we focused on three species of Acanthuridae (*Acanthurus nigricans, Ctenochaetus cyanocheilus,* and *Ctenochaetus marginatus*) and three scarine labrids (*Scarus frenatus, Scarus oviceps,* and *Chlorurus spilurus*). These six species are known to graze on CCA and are among the most common at Palmyra in terms of both abundance and biomass (Hamilton et al. 2014). Fish observations were conducted at the same four sites as the benthic community and CFD surveys (FR3, FR5, FR7, and FR9), although not directly at previously observed fungal concentrations, to avoid bias favoring observing CFD-targeting behavior. Two divers selected individual adult fish, as determined by phase coloration and size, for 3–6 min, usually from 2 to 4 m away to minimize behavioural disturbance (visibility generally exceeded 8–10 m). Species ID and estimated length were recorded for each fish, and substrate targeted by each bite was categorized as: scleractinian coral, CCA, mixed turf algae, *Galaxaura* spp., *Dictyota* spp., *Halimeda* spp., *Lobophora* spp., coralline lethal orange disease (CLOD), coralline fungal disease (CFD), or other. These data were summarized post hoc into five primary edible substrate types to correspond directly the summary categories of the benthic community substrate surveys: live hard coral, CCA, dead coral/rubble/turf algae, macroalgae, and CFD (there were no observations of CLOD). Bite rates are reported as mean bites per minute for each substrate type and fish species. Statistical analyses were performed using R (R Core Team 2016), unless otherwise noted.

To supplement observer data and to document species or ontogenetic stages other than the adult targets of the behaviour surveys, fish grazing was additionally monitored remotely by GoPro Hero 4 cameras with underwater color-corrective inserts (GoPro, Inc., San Mateo, CA, USA). These were affixed to the benthos with weighted bases between 10:00 AM and 4:30 PM approximately 0.5–1.0 m away from CFD fungal lesions, for ~ 1 h. Video was reviewed with observers recording species, estimated length, and targeted substrates for all fish interacting with the benthos within view of the lens. The video data were not used in the feeding preference analysis due to being focused on CFD lesions in the viewing frame (and thus excluding/reducing observations of grazing on all other substrates), but were instead used to confirm the a priori species selections and development stages used in direct diver observations and to survey for other potential CFD consumers.

### Fish feeding preference analysis

From the benthic cover and disease surveys a total of five primary categories of substrates palatable to herbivorous fish were used for preference analysis, comprising 87.1% of the total observed benthic cover data (the five categories were live scleractinian coral, CCA, dead coral or coral rubble with turf algae, macroalgae, and CFD). This subset of five categories was proportionately rescaled to total 100%, to represent abundance of palatable substrates as relative percent, as required for calculation of a modified Manly’s Selectivity Index (Manly et al. 1972; Chesson 1983; Manly 1995), which indicates whether a selective preference for a specific forage item exists with respect to the relative availability of that food item (*n* = 5 in our case). The formula was modified sensu Hamilton et al. (2014), with availability of a given forage resource (*n*) considered static, and not recalculated after each individual grazing event to reflect altered substrate availability (based on bite size relative to resource abundance). Preferences were calculated as follows:

$${\alpha }_{i}=\frac{{r}_{i}/{n}_{i}}{\sum_{j=1}^{m}{(r}_{j}/{n}_{j})}, \quad (i-1,..., m)$$

where *α* = Manly’s Selectivity Index (range from 0.0 to 1.0), *r*_*i*_ = the total bite rate (bites min^−1^) on substrate type *i* (in this case, with five substrate types), and *n*_*i*_indicates the proportion of that substrate (of total edible substratum) available for consumption. Substrate-specific α were calculated for each fish species and for each of the two aggregated taxonomic groups (i.e. parrotfishes or surgeonfishes). The ‘null’ value for the index (*α* = 0.2 for the 5 targeted substratum types) indicates no directed preference among the substrate types, with relative proportion of bites evenly distributed across each substrate relative to the abundance of that substrate. Values of *α* < 0.2, therefore, represent avoided substrate types, while *α* > 0.2 represented preferred substrates.

Due to the wide distribution in proportion in availability of substrate (prey) types, and the known sensitivity of Manly’s Index to rare prey types (Confer and Moore 1987), substrate preference was also calculated using Strauss’ Linear Index (1979). Preferences were calculated as follows:

$$\mathrm{L}\mathrm{i}={R}_{i}-{p}_{i},$$

where Li = Strauss’ Linear Index, *R*_i_ = total bite proportion (i.e. proportion of total bites for that species on a given substrate; sum of all *R* = 1), and *p*_i_ = the proportion of that substrate (of total edible substratum; sum of all *p* = 1) available for consumption. Note that *R* is not a rate, but a proportion, different than *r* in Manly’s Selectivity Index calculations. Index range is from − 1 to 1, with positive values indicating preference and negative values indicating avoidance. In a null situation (preference equal to abundance) Li = 0 for all substrate types.

### CFD dynamics

#### Herbivore exclusion experiments

To experimentally evaluate effects of fish grazing on CFD lesion progression, six CFD lesions were fitted with in situ exclusion cages at two sites south of Palmyra at 10–12 m depth. Selected lesions were all approximately the same size (~ 2 cm in diameter). Cages consisted of a 35-cm diameter hemispherical stainless steel mesh dome (mesh size ~ 2.3 mm) fitted with a flexible nylon net (mesh size 2.5 cm) skirt (~ 50 cm in width) that allowed us to better secure the cage on the irregular coral substrate to ensure effective exclusion of large (> 1 cm long) free-swimming organisms (Online Resource 3). These rigid cages were used because of the high-energy environment in the open-ocean fore-reef environment, and the difficulty fixing long poles or large mesh structures. Cages were left in place for 13 days. An additional six independent CFD lesions of approximately the same size and in the region of the caged lesions (< 10 m away) were marked with cattle tags and left uncaged as controls. All CFD lesions, both caged and uncaged, were photographed before and after cage removal under ambient light, using a Canon PowerShot G7 20.1-megapixel digital camera in a flat-port underwater housing with a scale bar placed horizontally in the image. Images were manually analysed for total areal extent of CCA thallus and visible fungal infection with a semi-automated image segmentation analysis program (Neal et al. 2015) using Matlab V.2014a (The MathWorks 2014). Mean percent change per day in lesion area for both the caged and uncaged sets (*n* = 6 for each) was compared with *T* tests after confirming data normality.

In order to assess cage effects, HOBO Pendant temperature and light data loggers (Onset Computer Corporation, Bourne, MA, USA; Product # UA-002-08; accuracy ± SE 0.53 °C from 0° to 50 °C) were anchored vertically inside and outside of the mesh domes to record temperature and lux every five minutes on two of the caged treatments, with one set at each of the two sites (Electronic Supplemental Material #1). Light intensities (lux) were converted to photosynthetically active radiation (PAR) using the equation: 1 μmol quanta (400–700 nm) m s^−1^ = 51.2 lx (Valiela 2013), and daily light interval (DLI) was calculated by integrating the daily PAR measurements for the diel light period (0645–1900 local time), expressed in units of moles photons per square meter per day (mol m^−2^ day^−1^).

#### Long-term lesion outcome tracking

In order to make an a priori estimate of long-term outcome dynamics of CFD lesions, we established in 2016 a set of tagged and photographed CFD lesions (*n* = 16). We returned in 2017 to these lesions, tagged a year earlier. These 16 lesions were only observed, and not caged, sampled, or altered in any way. Cattle tags were placed ~ 50 cm from the active lesions to avoid creating physical disturbance to host CCA or CFD while tagging, and to ensure no hydrodynamic disturbance. These lesions were photographed and analysed for planar area similarly to the lesions included in the shorter-term caged and uncaged lesion experiment in 2017. After imaging these 16 lesion areas in 2017, four of the lesions (the only ones with remaining fungal lesion tissue visible on the surface) were also used in the caging experiment. This year-long tagging experiment was intended to gauge expectations for long-term lesion outcomes, but not to estimate shorter-term lesion growth rates, as there were only two time points. It was not possible to have the lesions photographed in the intervening year due to the remote location.

## Results

### Grazing preference

#### Benthic community composition

**Fig. 4:**
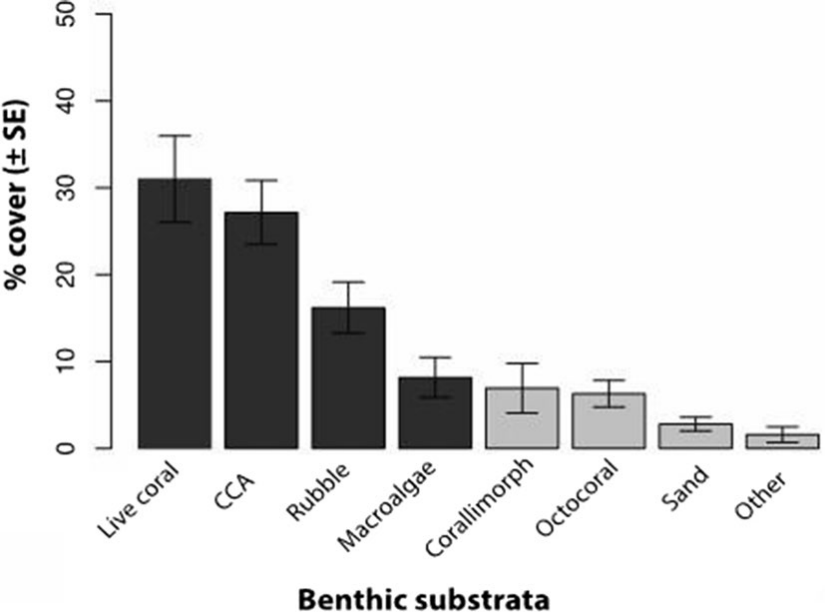
Summary of aggregate benthic community composition for all sites on the fore reef on Palmyra Atoll, in order of abundance. “Other” category includes *Fungiidae *ssp., manmade objects, and unidentified substrate. Black columns indicate the four primary targeted substrate types, along with CFD lesions, that were included in modified Manly’s Index feeding selectivity analysis

The mean cover across the atoll for our four primary benthic cover categories (hard coral, CCA, rubble/turf, macroalgae) was 31.0% ± 4.9 (SE hereafter), 27.1% ± 3.6, 16.2% ± 2.9, and 8.2% ± 2.3, respectively (Fig. 4). Reef builders, including live hard coral and CCA together, occupied 58.1% of the benthos, indicative of the healthy condition of this reef. Our observed percent cover for these categories were largely within the range of previously published studies for this location and reef habitat (Sandin et al. 2008; Williams et al. 2013; Gove et al. 2015).

#### In situ coralline fungal disease (CFD) surveys

A total of 12 CFD surveys were completed, three at each of the four sites. Transect coverage per survey varied from 150 to 590 m^2^, covering a total area for the study of 3987 m^2^, involving 10.6 h of active underwater observation time, with a mean survey area coverage rate of 6.3 m^2^ min^−1^. Coverage at each of the four sites was 1056, 1304, 1042, 585 m^2^, at sites FR3, FR5, FR7, and FR9, respectively. The reduced survey coverage at site FR9 was not intentional, but reflects difficult diving conditions at that location (high current). Mean linear travel while conducting the band transect survey was 3.05 ± 0.59 m min^−1^, ranging from 1.5 to 4.83 m min^−1^. A total of 902 fungal lesions were recorded in the surveyed area across the atoll with a mean density of 0.23 lesions m^−2^ of surveyed benthos or 1.11 lesions m^−2^ of CCA host, given our observed mean CCA coverage for each of the four sites.

Total combined lesion coverage, as calculated from maximum measured lesion diameter, was not greater than 1.43 m^2^ over a total of 3164 m^2^ of surveyed benthos (a total area roughly comparable to that of the benthic substrate surveys above, at 3987 m^2^), or 0.045% of total benthic area, with a mean lesion size of 16.25 cm^2^ (calculated from mean maximal diameter for all lesions = 4.55 cm). This percent cover estimate is almost certainly a slight overestimation due to our assumption of circularity in the calculation based on maximal diameter of a CFD lesion, and the observed irregularity of lesion shapes. Given the relatively small total lesion coverage, even during acute CFD outbreaks post El Nino, this marginal overestimation of total lesion coverage was retained in subsequent calculations of feeding preference, because it would ensure a slightly conservative outcome when estimating grazing preference for this category; marginally larger areal coverage estimate for any individual category would result in proportionately lower (i.e. conservative) calculation of relative feeding preference.

The disease conditions we recorded are similar to a previously observed late-stage fungal outbreak in this location. Our study took place immediately following a period of elevated sea surface temperatures, which is known to contribute to fungal outbreaks (Vargas-Angel 2010; Williams et al. 2014). The high densities of fungal lesions seen in 2017 (1.11 lesions m^−2^ of CCA host) were similar to lesion densities recorded during the 2009 El Nino (1.37 lesions m^−2^ of CCA host), compared to background levels of ~ 0.1 lesions m^−2^ of CCA host in non-outbreak conditions (Williams et al. 2014). Further evidence confirming that our observed lesion densities were elevated for the region is provided by Vargas-Angel (2010), recording CFD lesion densities across a wider range of the Central Pacific (42 locations) during the 2009 CFD outbreak, with our observed lesion occurrence in Palmyra exceeded by only two other locations (Tutila and Ofu-Olu in American Samoa).

#### Herbivorous fish behaviour surveys

**Fig. 5:**
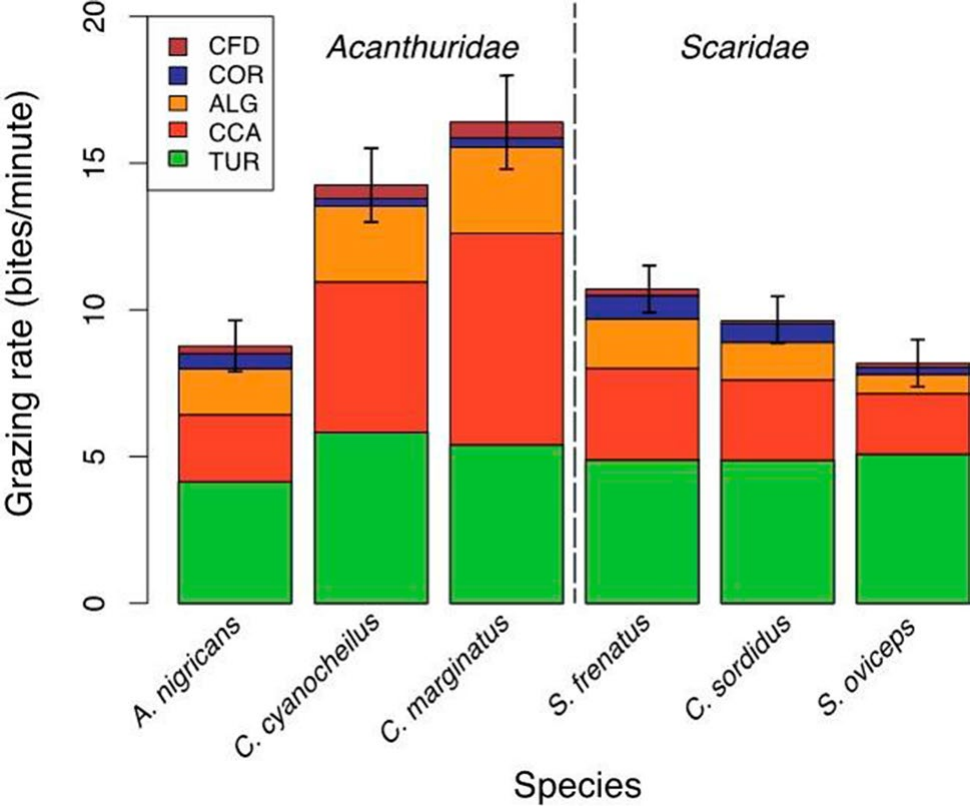
Substrate grazing selection by species, with vertical dashed line separating the two primary taxonomic groups (Family *Acanthuridae* and scarine labrids*)*. *TUR* turf algae, *CCA* crustose coralline algae, *ALG* other macroalgae, *COR* hard coral, *CFD* coralline fungal disease. Error bars indicate SE for bite rates observed per species (for all substrates)

A total of 191 fish (101 Acanthuridae and 90 scarine labrids) were observed in 2016 over a total of 734 min averaging 3.8 min of behavioural observation fish^−1^ (Table 1). A total of 8491 fish bites were recorded, at a mean bite rate of 11.6 bites min^−1^. There was no significant difference between bite rates of the three Acanthuridae species (13.4 ± 2.3 bites min^−1^) and the three species of 90 scarine labrids (9.6 ± 0.7 bites min^−1^) (*p* = 0.183, *t* = 1.61, *df* = 4) (Fig. 5). Observed bites on the five primary substrate types specified in the benthic community and CFD surveys accounted for 98.5% of all bites, so a scaled adjustment to the bite dataset was not made, as it was for the benthic substrate data, to correct for this minor level of bites on uncounted substrate types (Table 2). Bites on CFD accounted for 2.48% of observed bites, while CFD itself only occupied 0.03% of the total substrate.

**Table 1:** Summary of species, sample sizes, and observed mean total length for fish observed for feeding behaviour in this study

Species	*N*	Mean total length, cm (± SE)	Mean forereef biomass, g m^−2^ (± SE)	Mean forereef abundance, indiv. m^−2^ (± SE)
*Acanthurus nigricans*	36	15.0 (0.6)	**8.5 (1.9)**	**0.08 (0.02)**
*Ctenochaetus cyanocheilus*	30	13.4 (0.5)	**5.0 (1.6)**	**0.07 (0.02)**
*Ctenochaetus marginatus*	35	18.3 (0.6)	**3.8 (0.7)**	**0.03 (0.01)**
Surgeonfish (all)	101	15.6 (0.3)		
*Scarus frenatus*	25	23.9 (0.8)	**1.7 (0.4)**	**0.01 (0.00)**
*Scarus oviceps*	26	20.7 (0.5)	**0.3 (0.2)**	**0.00 (0.00)**
*Chlororus spilurus*	39	24.5 (0.8)	**11.68 (1.6)**	**0.04 (0.01)**
Parrotfish (all)	90	23.0 (0.4)		

Previously published biomass and abundance estimates for these groups on Palmyra Atoll (Hamilton 2014) are also shown (bold value columns), as used in “Discussion” for comparison of relative potential impact of these herbivorous groups on lesion control

**Table 2:** Summary of substrates targeted in observed fish bites and benthic cover for Palmyra Atoll, July 2017

Benthic substrate type	% of bites	% observed benthic cover	% corrected benthic cover
Turf algae (inc. turf on broken and dead coral substrate)	43.30	16.17	19.6
Crustose coralline algae (CCA)	33.12	27.14	32.93
Macroalgae (inc. *Galaxura, Halameda, and Lobophora* sp.)	15.67	8.15	9.87
Live hard coral	4.01	30.98	37.57
Coralline fungal disease (CFD)	2.48	0.03	0.03
Other	1.42	15.08	0
Total	100.00	100.00	100.00

The centre column indicates actual measured benthic cover, and the right-hand column indicates corrected relative abundance of the five primary substrates, as used for Manly’s selectivity analysis calculations

#### In situ video fish feeding observations

During 13.8 h of video, 39 individual fish were observed closely interacting with the area of the fungus, with 15 of these taking bites directly on the lesion area (Online Resource 4). A total of 315 bites were observed, with 62 of those directly on the CFD lesion, and the remainder on other substrates within the video frame. Thirty-three of the 39 fish observed were our three target species of Acanthuridae. Among these surgeonfish were juveniles, an age class which was not observed in the individual fish behavioural observation data set collected by SCUBA divers. Interestingly, no scarine labrids of any species were recorded on video feeding near fungal lesions. An additional four species (totalling 6 fish) were seen taking a total of 12 bites near CFD lesions (Electronic Supplemental Material #2).

#### Fish feeding preference analysis

**Fig. 6:**
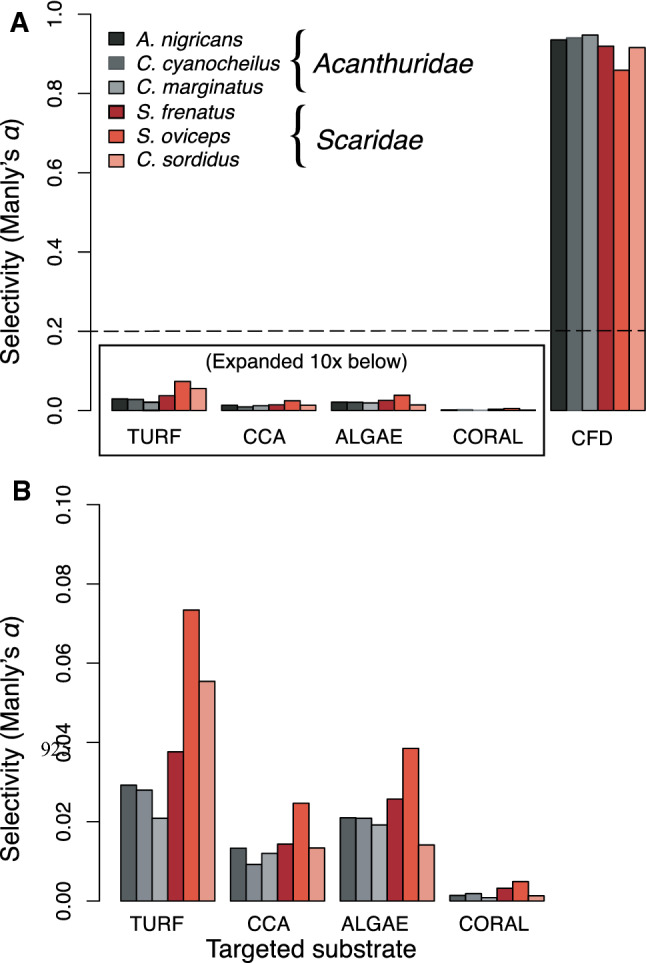
Manly’s Selectivity Index showing grazing preferences for all herbivores observed. **a** Index scaled to 0.0–1.0 for all benthic substrate categories, showing strong selection preference for CFD lesions, when present. Surgeonfish are shown in black gradations and parrotfish in red. Dotted horizontal line indicates ‘null’ preference assumption (*α* = 0.2), with equal grazing of all categories. **b** All categories except CFD scaled 0.0–0.1, to expand most common substrate selections. These four substrata accounted for 97.5% of all observed bites. The relative avoidance of live hard coral, the most common substrate in this system, was seen in all species observed

The modified Manly’s Selectivity Index showed that all six fish species strongly targeted CFD lesions (Fig. 6a). Under the null model (i.e. no preference in fish feeding behaviour by substrate) each substrate would be targeted relative to abundance, and the respective Manly’s index would be 0.2 for all five substrates (summing to 1.0). Manly’s selectivity scores > 0.8 for CFD for all six species indicate a strong preference for this substrate, when available. The other four non-CFD infected edible substrate types were all below 0.1 on this scale suggesting that relative to CFD, these substrate types are not as preferred. Among these non-CFD-infected edible substrate types, turf algae was the most preferred category and live hard coral least preferred (Fig. 6b). Our findings of proportional preference for the four non-CFD substrates were largely in agreement with previously published grazing target ratios for these substrates on Palmyra and other tropical Pacific reef environments (Hamilton et al. 2014).

Li index values for the five substrate types indicated a strong avoidance of hard coral substrate (negative value), and a preference CFD (positive values) for turf, CCA, macroalgae, and (Table 3). Li values decrease in magnitude with the relative abundance of prey items in the environment, and thus underestimate selectivity for rare items, but a positive or negative assignment for any given calculation will reflect avoidance or preference of that item. This index indicates that interactions between fish and CFD lesions are not simply random events, but are proportionately more common than would be expected from chance encounter alone and more than threefold higher than that of uninfected coralline algae.

**Table 3:** Strauss’ Linear Index of Food Selection results, using corrected relative benthic cover for substrates of interest (summing to 100% for calculation purposes)

Substrate	% relative benthic cover	*R*_i_	*P*_i_	Li
*Turf algae*	19.60	0.439	0.196	0.243
*CCA*	32.93	0.336	0.329	0.007
*Macroalgae*	9.87	0.159	0.099	0.060
*Live hard coral*	37.57	0.041	0.376	− 0.335
*CFD lesions*	0.03	0.025	0.0003	0.025

Substrate types are presented in same order as Table 2, (also using substrate proportions scaled for calculations). CFD *P*_i_ shown to four decimal places to reflect rare nature of this substrate, all others to three places. This index is presented primarily to indicate that a selective preference exists for all substrates with positive values, but note that for rare substrates (like CFD) the Li value may under-represent actual foraging selectivity

#### Herbivore exclusion experiments

Five of the caged lesions (at two sites) remained in place and appeared successful in excluding herbivorous fish for the duration of the experiment, as evidenced by absence of new feeding scars on the CCA within the caged area. Mean initial lesion size was 1.86 cm^2^ (± 0.37) and did not differ significantly between caged (1.97 ± 0.69 cm^2^) and control (1.77 ± 0.34 cm^2^) (*p* = 0.091, *t* = 0.11, *df* = 9) (Table 4). The difference in mean temperature between treated (caged) and untreated (open) at both sites was less than the accuracy of the HOBO Pendant monitoring instruments (± 0.53 °C), indicating no measurable treatment effect. Mean water temperature was 29.32 (± 0.0)°C at site FR3 and 29.27 (± 0.01) °C at site FR5. There was a 69.7% reduction in total PAR under the cages compared to the uncaged controls, but the daily PAR levels within the caged treatments remained above thresholds where CCA growth may be impacted (**< **0.1 mol photons m^−2^ day^−1^; Bessell-Browne et al. 2017). The mean daily light integral (DLI) for the measured treatments (*n* = 2) was 1.7 and 0.5 (± 0.3 and 0.2, respectively) mole photons m^−2^ day^−1^, outside and inside the treatments, respectively.

**Table 4:** Individual fungal lesion initial and final sizes, and percent change in size over the observation period, for both uncaged (*n* = 6) and caged (*n* = 5) treatments

Treatment	Uncaged lesions			Caged lesions		
	Initial (cm^2^)	Final (cm^2^)	% change	Initial (cm^2^)	Final (cm^2^)	% change
1	2.57	0.40	− 84.5	1.31	9.26	605.0
2	2.52	1.38	− 45.1	1.59	4.37	175.6
3	2.41	3.99	65.6	1.15	5.71	395.2
*4*	0.47	0.39	− 16.6	4.72	11.36	140.5
5	1.34	1.82	36.3	1.07	19.11	1682.1
6	1.32	1.46	10.3			
Mean size (cm^2^)	1.77	1.57		1.97	9.96	
Median size change			− 3.1			395.2

**Fig. 7:**
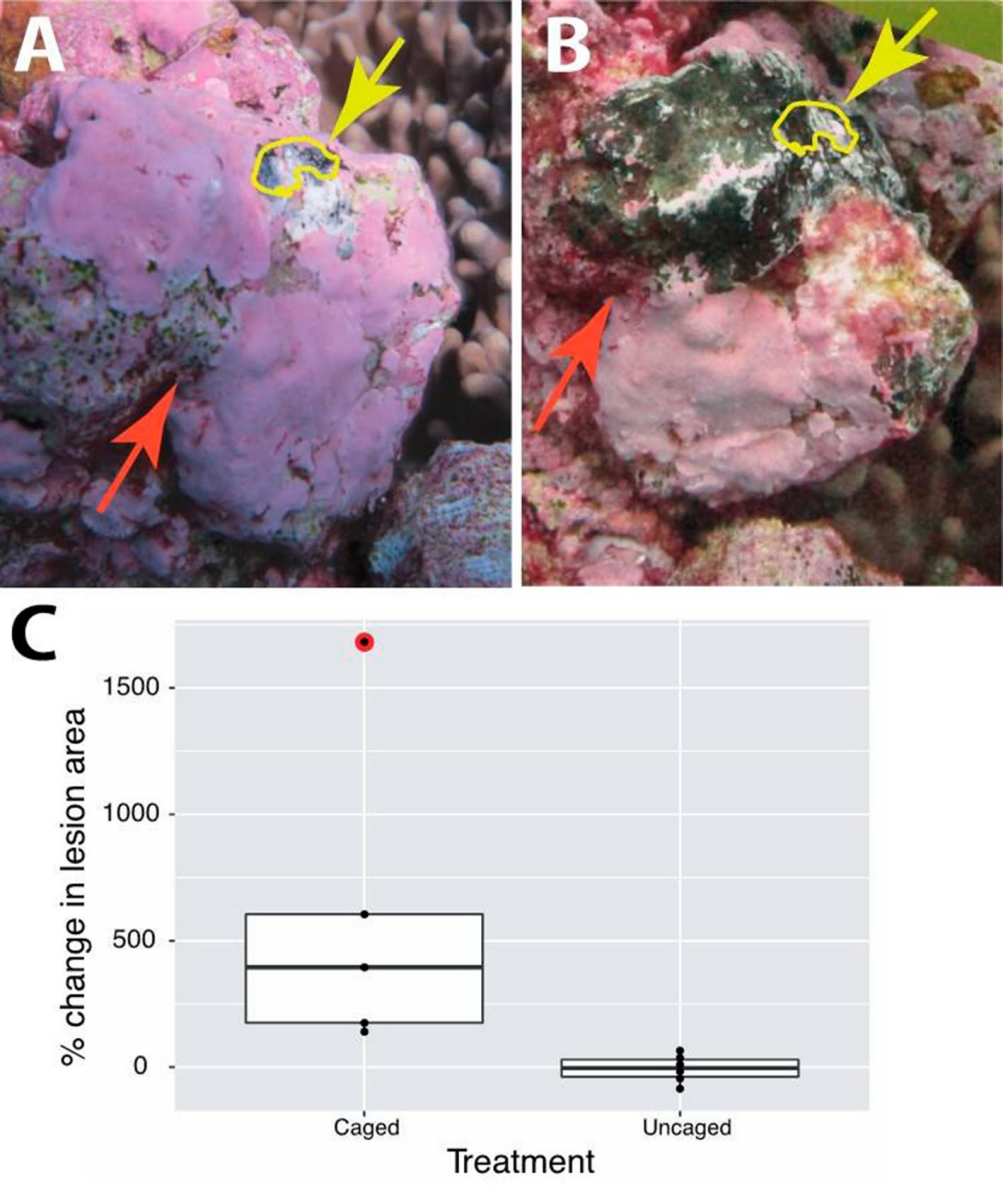
The largest example of expansion of fungal lesion area in a caged lesion experiment (Cage #6) over the study period, from **a** initial condition, to **b** final condition. Yellow arrows and yellow polygon indicate the approximate dimensions of the original lesion area in both images, with the red arrow indicates a reference landmark within the area of primary lesion expansion (note that perspective varies slightly in image series). **c** Percent changes in caged (*n* = 5) and uncaged (*n* = 6) lesion treatment sets over the study period. Median lesion change in the uncaged treatment was − 3.1%, and median change in the caged treatment was 395.2%. Box plot indicates median, first and third quartiles, and all points (black dots). The red symbol indicates the change observed in Cage #6, which had the largest relative expansion of any caged lesion, as shown in **a**, **b** above

Mean lesion expansion for all five caged CFD lesions over the course of the 13 days was 599.7% (± 283.2) ranging from 140.5 to 1682.1%, with a mean daily planar area expansion of 58.2 mm^2^ day^−1^ (± 18.9), ranging 21.3–128.84 mm^2^ day^−1^ (Fig. 7). Mean size change in the uncaged lesion set (*n* = 6) was statistically unchanged for the set over for the course of the experiment (− 5.6% ± 22.4%), but individual lesion change for the uncaged set over the duration of the experiment was highly stochastic, ranging from + 65.6 to − 84.5% of original area (Table 3), and mean daily planar area expansion for these lesions were similarly variable and not different from zero (mean − 1. mm^2^ day^−1^, ± 3.9; *p* = 0.69, *t* = 0.41, *df* = 10), ranging from − 16.6 to 11.3 mm^2^ day^−1^. New bite marks from herbivorous fish were clearly visible on some of the uncaged lesions. The median rate of lesion expansion in caged CFD significantly exceeded that of uncaged (*p* = 0.042, *t* = 2.35, *df* = 9).

#### Long-term lesion outcome tracking

In July 2017 12 of the 16 (75%) lesions tagged for observation in July 2016 (1 year prior) showed no visible sign of fungal infection. Nine of these areas returned to live CCA cover, one to encrusting scleractinian cover, and two to mixed turf algae. Four areas of visible fungal infection remained of the original 16, with one of these located in an area adjacent to (~ 17 cm away), but not directly covering the area of the original lesion, and the rest occurring on the original area of infection. Given our incomplete understanding of both fungal hyphae penetration into the calcium carbonate substrate as well as new lesion establishment mechanisms (i.e. through waterborne spores or tissue transfer), this individual lesion may or may not be directly related to the original lesion. We assumed for the remaining three lesions, all located on part of the original area, that these were remnant portions of the original lesions. Mean lesions size for of the remaining four lesions in 2017 was 2.17 cm^2^^1^ (± 4.02), compared to 11.85 cm^2^ (± 0.86) for those same lesions in 2016, a significant decline in size for these surviving lesions (− 81.6%; *p* = 0.04, *t* = 2.52, *df* = 6). Three of the remaining four lesions were very small, and had no visible fish grazing scars, and one (the only remaining lesion presenting a large surface area) showed fish grazing scars that had occurred since the last photo.

## Discussion

Selected herbivorous reef fish may be preferentially grazing on superficial fungal mats on CCA and experimental exclusion trials suggest that release from grazing by herbivorous fish may contribute to more rapid progression of CFD lesions. Mycophagy by herbivorous fish may therefore serve to impact fungal disease dynamics in coral reef systems through direct removal of the fungal pathogen. Preferential mycophagy by herbivorous fish has not, to our knowledge, been previously documented in tropical systems, and is of interest from both the organismal perspective as well as how it relates to CFD disease dynamics. Marine mycophagy could affect CFD dynamics and affect overall virulence of the disease through multiple factors; the strongly targeted consumption of fungal lesions may have a top-down effect, reducing infected area and preventing or delaying hyphal progression and expansion, or grazing and removal may also be controlling CFD progression through reduction of fungal fecundity and spore release.

The potential relationship between fungal lesions and herbivorous fish may extend the ecological importance of reef fish as a community stabilizing force in coral reef ecosystems, showing that an extant and healthy herbivorous fish population can not only act in their well-documented roles as grazers limiting overgrowth of reef-building organisms by fleshy algae, but also as potentially important ecological components of disease control for primary producers such as CCA that form the foundation of coral reef substrata. Higher reef fish abundance and diversity are inversely related to overall scleractinian coral disease prevalence within a reef system (Raymundo et al. 2009), and our findings potentially extend our understanding of the ecological role of herbivorous fish on tropical reefs to include interactions with fungal diseases affecting CCA.

We can quantitatively estimate the biological control impact that herbivorous fish may exert on CDF outbreaks, using our measurements of fungal lesion abundance, lesion growth rates, our observed rate of grazing by herbivorous fish on fungal lesions, previously estimated abundances of herbivorous fish at this location (Hamilton et al. 2014) and making a series of assumptions. These estimates, calculated below, allow us to speculate on the ability and role of fish herbivory to supress overall potential spread of CFD in this system. During the CFD outbreak, we observed a mean density of 1.11 lesions m^−2^ of CCA host, with a mean size of 15.54 cm^2^ per lesion, giving a total mean measured extent of 17.25 cm^2^ of CFD lesion m^−2^ of CCA host. Multiplying this estimate of CFD area per host by our observed mean CCA host coverage on the benthos of 27.1% (e.g., 0.271 m^2^ of CCA per m^−2^ of reef benthos) yields an estimated area of 4.67 cm^2^ of CFD lesions m^−2^ of reef bottom (~ 0.05% CFD cover of benthos). To estimate possible population growth from this level of reef-wide CFD infection in the absence of grazers, we use the mean lesion expansion rate as measured in our herbivory exclusion experiment, which yielded approximately 600% mean lesion expansion over the course of the 13 days, corresponding to an exponential growth rate of 0.16 day^−1^. In the case of our observed density as stated above, this would result in growth of 2.51 cm^2^ of CFD lesion m^−2^ of CCA host day^−1^, or 0.68. cm^2^ of CFD lesion m^−2^ of reef benthos day^−1^. To contrast this to potential herbivorous grazing pressure, we used our mean bite rate for all species of 11.6 bites min^−1^ along with our observed target rate on CFD of 2.48%, to result in an estimated 0.29 bites min^−1^ on CFD per fish. If we assume each fish is fully active for only 8 h per day, this results in an estimated 139.2 bites on CFD per fish each day. Using a previously measured density on the fore reef in Palmyra for the combined guilds of parrotfish (0.05 individuals m^−2^) and surgeonfish (0.18 individuals m^−2^) (Hamilton et al. 2014), gives a total of 0.23 individual herbivorous fish m^−2^ of fore reef habitat. Assuming no overlap of bites, we multiply the density of fish and bite rate by an estimated fish bite size of 1.5–5 mm^2^ bite^−1^ (Bellwood and Choat 1990) to estimate total potential removal of CFD of 0.48–1.44 cm^2^ of lesion m^−2^ of reef benthos day^−1^. To summarize, we estimate potential growth of 0.68 cm^2^ of CFD lesion m^−2^ reef benthos day^−1^ in outbreak conditions is within the range of estimated removal rates (0.48–1.44). Noting that the upper estimation removal rate is well over double the growth rate, these rough estimates support the concept that mycophagy by herbivorous fish could exert a controlling and limiting effect on expansion of the fungus. Furthermore, it is possible that the targeted grazing rate on CFD will change (i.e. increase) as the fungus becomes more abundant. However, it also clear from these idealized calculations that substantial healthy adult herbivorous fish populations are required to tip the balance in favour of CFD removal over CFD expansion.

The above estimates of lesion growth and CFD disease dynamics assume the existence of pristine herbivorous fish populations, and assume that our observations of lesion growth rates are static. However, neither of these assumptions may be true in many reef systems in the near future as a result of both global change (Hoegh-Guldberg and Bruno 2010) and ever-increasing fishing pressure (Jackson et al. 2014). Conditions which deplete the population of grazing fishes on a reef, change the population size structure and thus grazing rates or physical gape sizes (Zglicynski and Sandin 2017), or those which either increase the rate of spread of the fungus or reduce the coverage or infectious resistance of CCA could all potentially alter the progression and severity of CFD. Our estimates of removal of CFD by grazing suggest that if fish populations are reduced by more than ca. 50%, as they are in many reef systems, then the balance of ecological control could change. Future studies of the dynamics of this disease could examine CFD prevalence in heavily fished versus unfished reefs to confirm whether or not grazing fish are in fact having the controlling effect of reducing CFD and decreasing impact to CCA as suggested here.

In contrast to the beneficial effects for CCA that reef fish may have by reducing prevalence of CFD, there is the possibility that the action of herbivorous fish may also be having an adverse effect on CCA by serving as vectors of fungal pathogens to new uninfected crusts. Evidence in support of this idea is that Palmyra, with no fishing and a large extant herbivorous fish population, still has significant CFD infection relative to other Pacific locations (Vargas-Ángel 2010). One possible explanation for this paradox is that herbivorous fish mouthparts may also be acting to transport fungal hyphae to new locations, or that fungal hyphae or spores are being passed through the gut and deposited by defecation. A similar mechanism was proposed for cyanobacterial disease in corals thought to be transmitted by feeding activity of butterflyfish (Aeby 2006). Further work to test this hypothesis could include examining mouthparts or gut contents for genetic evidence of the fungus, or using simulated mouthparts to attempt to initiate new infected locations. Another potentially adverse effect stemming from fish grazing could be that the scars left by the scraping of mouthparts may provide routes of entry for waterborne fungal spores or hyphae into CCA tissues. Similar mechanisms are seen for certain fungal diseases of terrestrial plants (Agrios 2005).

CFD may also not depend on fish as vectors but could simply be spread by waterborne spores or direct contact (McCallum et al. 2003). Finally, CFD could be spread by other organisms that feed on CCA including cryptofauna such as limpets (Steneck 1982), urchins (Bulleri et al. 2002), and gastropods (Paine 1980), the last of which is a known vector for coral disease (Williams and Miller 2005). We did not see direct evidence in the video footage or in the field of the action of these community members, but cryptofauna are often crepuscular (we did not video at dawn or dusk) and their role as potential CFD infection vectors or consumers of the fungus merits additional study. Further work should utilize a variety of exclusionary devices, and (if possible) gut content analysis of potential invertebrate vectors, to obtain a clearer understanding of trophic interactions and the impacts that the rest of the coral reef community may be having on CDF dynamics.

While the cage exclusion experiment seemed to support the concept that lesions may be controlled by continuous direct consumption, caution must be exercised in ascribing the observed positive result only to release from grazing. The design of the exclusion devices did not exclude all possible grazers, specifically small benthic invertebrates capable of using internal access routes through the substrate, and furthermore may have altered the local environment inside the cages to favour CFD expansion. Specifically, one alternative explanation for our experimental observations may be localized alteration of the hydrodynamic or thermal environment within the cages. Light levels in particular were observed to be significantly reduced within the caged treatments due to the wire mesh of our cages. However, our recorded light values are likely an underestimation of the DLI as the HOBO pendants were hindered in their vertical orientation by the installation within the cage, and thus were possibly under-sampling available light. If light was actually lower in the cages, this may have caused a reduction in CCA resistance to infection, and advantaged fungal overgrowth. However, the impacts from this light reduction are uncertain given that the available light was still above the minimum threshold for CCA growth, but is presented as one possible confounding factor in interpreting the dramatic fungal growth observed in the cages. Notably, little or no fungal infection was seen in shaded or protected areas of the natural reef, with the undersides of tabular corals thickly covered in healthy CCA crusts, with no observed fungal infections in these perpetually shaded areas. However, the lack of observed infection in these areas may be because the sole known host for CFD (*P. onkodes)* prefers open, high-energy, high-light locations. Further work should include uninfected cage controls on a variety of substrates and exposures, as well as improved instrumentation and cage setup (i.e. open cage controls) to determine if there are confounding environmental impacts from exclusionary devices. Nonetheless, the observation from this exclusion experiment does suggest that fungal growth patterns can be highly dynamic on the time frame of just a few days, which is especially compelling given that the year-long observation of 16 tagged lesions in natural (i.e. exposed) conditions was largely static or declining, with 75% of previously tagged lesions disappearing and the remaining four lesions experiencing significant declines in size. It is notable that caging did appear to dramatically alter this trajectory of slow lesion decline or control, and supports future work investigating the mechanisms behind this dramatic growth pattern.

What may drive fish to preferentially consume fungi remains unknown. Fungi produce a variety of secondary metabolites (Keller et al. 2005), and it is possible that the herbivorous fish may be seeking these compounds. Some molluscs, for example, feed preferentially on fungal-infected tissues on terrestrial plants (Ramsell and Paul 1990); however, other compounds are produced by fungi to actively discourage grazing (Rohlfs 2015). Without a profile of the metabolites produced by the pathogen in this case, it is not possible to say if there is a positive or negative allelopathic influence on herbivory. Future isolation and culturing of the fungus could provide a reliable source of material for analysis of bioactive or nutritional compounds that may be driving the grazing behaviour. Alternatively, the observed targeted mycophagic behaviour could be in response to protein limitation. Many fungi contain significant protein concentrations (Ahmed et al. 2017), and parrotfish feeding has recently been hypothesized to be driven by microphagy targeting protein-rich cyanobacteria and other epithilic, endolithic, epiphytic or endosymbiotic microrganisms (Clements et al. 2017). The fish may thus be targeting CFD lesions not because of secondary metabolites, but simply to fulfill primary energetic needs. In contrast to the seemingly obvious concept that herbivory on superficial fungal lesions is targeting the fungal tissue themselves, it is also possible that the fish are alternatively taking advantage of the increased physical palatability of the softened CCA tissue following partial digestion by the overlying saprophytic fungi. Fish may be taking advantage of chemical or physical changes caused by fungal infection in the host tissue to access nutritive components otherwise entrapped in the carbonate crust.

If our observed mycophagy by herbivorous fish is in fact acting to suppress the impact of CFD on the crustose coralline algal community, this is a previously undescribed mechanism affecting overall reef health, function, and resilience. The widespread depletion of herbivore populations currently in many coral reef systems from overfishing may increase the risk of uncontrolled or damaging CFD outbreaks. The control of fungal infection could thus be an additional factor in motivating conservation action for herbivore protection, supporting the establishment of area closures such as Marine Protected Areas (MPAs), directing management policies such as size, species limits or gear limits on harvest, or encouraging active restoration of these herbivorous species (e.g., the Kahekili Marine Reserve in Maui, HI, USA) (Williams et al. 2016). In addition, with prevalence of CFD positively linked to warmer water (Vargas-Angel 2010; Williams et al. 2014), the potential contribution by herbivorous fish in controlling this disease is of increased importance in light of projections that climate change will increase thermal stress on reefs worldwide (Van Hooidonk et al. 2016). Given the fundamental importance of CCA to coral reef ecosystems as an ecosystem engineer (Birrell et al. 2008) and a substrate for coral recruitment (Diaz-Pulido et al. 2010) and disease impacts on this facilitative role (Quéré and Nugues 2015), managing healthy fish populations to control fungal disease could be influential in maintaining stable community structure in tropical coral systems. Our study adds the role of mycophagy by herbivorous fish in controlling fungal disease in CCA to the growing list of reasons why herbivores are critical for maintaining ecosystem balance on coral reefs, with a diverse and robust fish assemblage on coral reefs increasing resistance and resilience to the myriad stressors these ecosystems increasingly face.

## Electronic supplementary material

Below is the link to the electronic supplementary material.

Supplementary file1 (DOCX 2305 kb)

Supplementary file2 (DOCX 22 kb)
