# Supplementary material for: Possible control of acute outbreaks of a marine fungal pathogen by nominally herbivorous tropical reef fish
Source: Oecologia. 2020 Jul 12;193(3):603–17. doi: 10.1007/s00442-020-04697-7 (PMC7406524; doi:10.1007/s00442-020-04697-7)
Supplement: Supplementary file 1 — Supplementary file1 (DOCX 2305 kb) [file 442_2020_4697_MOESM1_ESM.docx]

**Electronic Supplemental Material 3**

**For:** Tropical reef fish herbivory control of acute outbreaks of a marine fungal pathogen

**AUTHORS^[[1]](#footnote-1)^:** Neal, BP^1^*; Honisch, B^1^; Warrender, T^2^; Williams, GJ^2^; Work, TM^3^; Price, NN^1^

***Corresponding author:** [bneal@bigelow.org](mailto:bneal@bigelow.org)

^1^ Bigelow Laboratory for Ocean Sciences, 60 Bigelow Drive, East Boothbay, ME USA 04544

^2^ Bangor University School of Ocean Sciences, Bangor University, Anglesey, UK


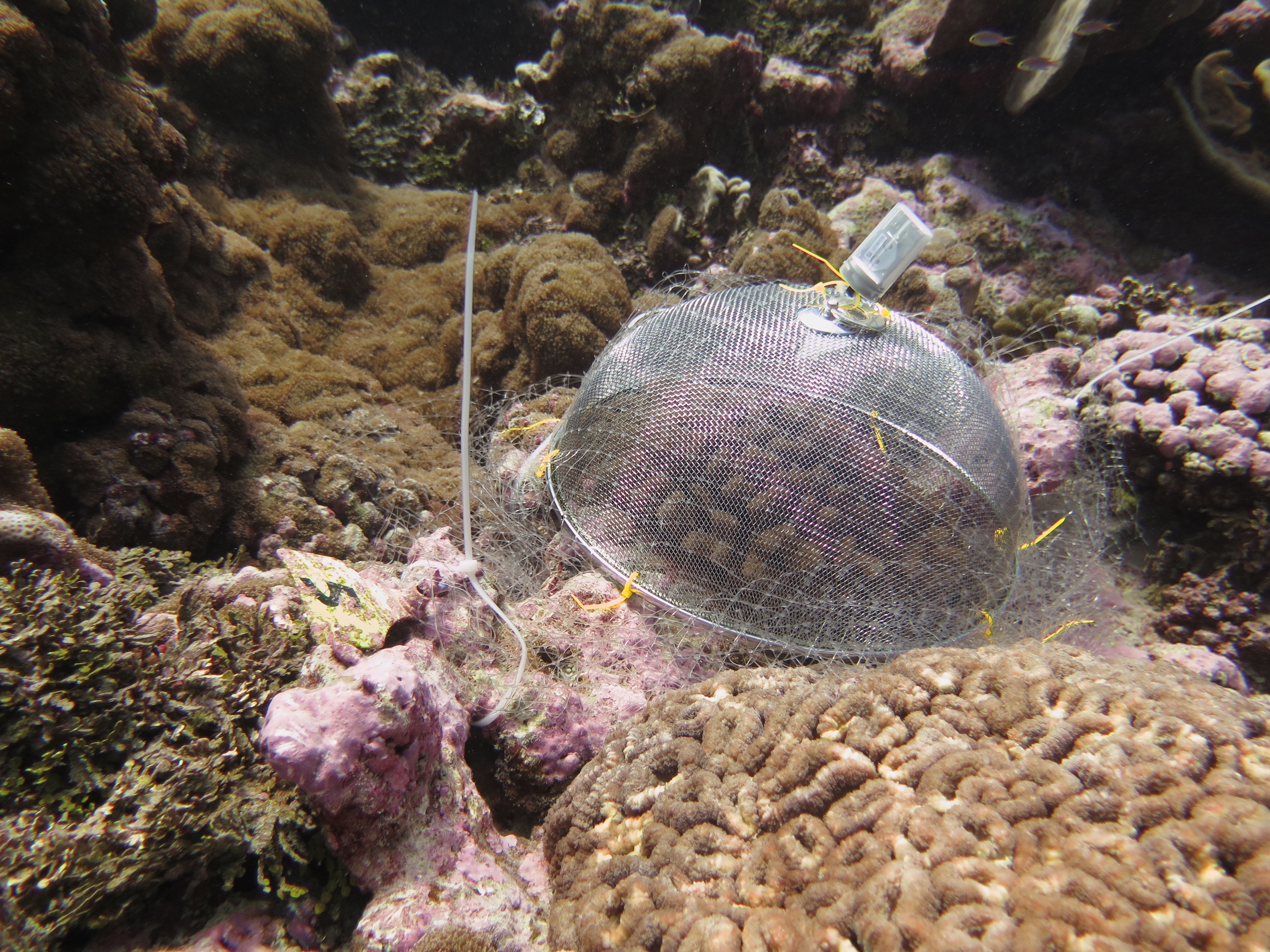
^3^ USGS National Wildlife Health Center, Ala Moana Blvd, Honolulu, HI USA 96850

Herbivore exclusion cage. The stainless cage is surrounded by visible netting skirt, fit and secured to the irregular substrate. Large herbivorous fish (>10cm) were clearly excluded from the caged lesion, but it is possible that small fish living in the substrate (e.g. small Blennidae, or Labridae) or mobile benthic invertebrates could have accessed the lesion of interest, but there was no observed evidence of this. HOBO temperature and light meters were placed inside and out as well, to assess possible cage effects.

1. [↑](#footnote-ref-1)
