# Supplementary material for: Possible control of acute outbreaks of a marine fungal pathogen by nominally herbivorous tropical reef fish
Source: Oecologia. 2020 Jul 12;193(3):603–17. doi: 10.1007/s00442-020-04697-7 (PMC7406524; doi:10.1007/s00442-020-04697-7)
Supplement: Supplementary file 2 — Supplementary file2 (DOCX 22 kb) [file 442_2020_4697_MOESM2_ESM.docx]

**Electronic Supplemental Material 4**

**For:** Tropical reef fish herbivory control of acute outbreaks of a marine fungal pathogen

**AUTHORS^[[1]](#footnote-1)^:** Neal, BP^1^*; Honisch, B^1^; Warrender, T^2^; Williams, GJ^2^; Work, TM^3^; Price, NN^1^

***Corresponding author:** [bneal@bigelow.org](mailto:bneal@bigelow.org)

^1^ Bigelow Laboratory for Ocean Sciences, 60 Bigelow Drive, East Boothbay, ME USA 04544

^2^ Bangor University School of Ocean Sciences, Bangor University, Anglesey, UK

^3^ USGS National Wildlife Health Center, Ala Moana Blvd, Honolulu, HI USA 96850

| **Species** | **Family** | **# observed** | **Total bites** | **Bites on CFD** | **Bites on CCA** | **Bites on other substrates** |
| --- | --- | --- | --- | --- | --- | --- |
| ***Ctenochaetus cyanocheilus*** | **Acanthuridae** | **17** | **148** | **19** | **11** | **118** |
| ***Ctenochaetus marginatus*** | **Acanthuridae** | **10** | **119** | **26** | **15** | **78** |
| ***Acanthurus nigricans*** | **Acanthuridae** | **6** | **16** | **5** | **6** | **5** |
| *Centropyge flavissima* | Pomacanthidae | 3 | 8 | 6 | 2 | 0 |
| *Gomphosus varius* | Labridae | 1 | 5 | 0 | 5 | 0 |
| *Cirripectes virulosa* | Blenniidae | 1 | 13 | 4 | 8 | 1 |
| *Cirripectes spp* | Blenniidae | 1 | 6 | 2 | 0 | 4 |
|  |  |  |  |  |  |  |
| *Totals* |  | 39 | 315 | 62 | 47 | 206 |

Species observed in video survey exhibiting benthic herbivory near target CFD lesions. The bolded species (n=3) were those for which behavioural follows were done, and the remaining four species were ones that were not identified *a priori* as potential species interacting with the fungal lesion. The Other category was largely composed of bites on turf algae surrounding the infected CCA crust, which was in keeping with earlier observations of turf as the most commonly targeted substrate. Note that the percentage of bites on CFD (19.7%), and on CCA (14.9%) are not representative of overall grazing patterns in this environment, because the camera was fixed on a limited area surrounding the target lesion, so behaviour recorded only reflects grazing in that immediate area.

1. [↑](#footnote-ref-1)
